# Stress and Support Present in Medical Crowdfunding in Pediatric Cancer

**DOI:** 10.1002/cnr2.70375

**Published:** 2025-10-19

**Authors:** Mary K. Killela, Sandra Garcia, Jessica Keim‐Malpass, Sandra Soto, Todd Schwartz, Jessica Williams, Sheila Santacroce

**Affiliations:** ^1^ Huntsman Cancer Institute, University of Utah Salt Lake City UT USA; ^2^ College of Nursing, University of Utah Salt Lake City UT USA; ^3^ School of Nursing, University of North Carolina at Chapel Hill Chapel Hill NC USA; ^4^ School of Medicine, University of Virginia Charlottesville VA USA; ^5^ Gillings School of Global Public Health, University of North Carolina at Chapel Hill Chapel Hill NC USA

**Keywords:** childhood cancer, cost of illness, financial burden, financial support, social networking, social support

## Abstract

**Background:**

Medical crowdfunding (MCF) seeks to mitigate the financial harm to families with a child with cancer.

**Aims:**

We sought to describe the illness phases, campaign creators, and the main topics discussed on MCF pages.

**Methods and Results:**

Using publicly available campaigns on GoFundMe.com, we randomly selected 100 campaigns to extract information on stressors, appraisals, and additional attributes. We conducted descriptive statistics to describe the distributions of illness phases, creators, and topics, and Fisher's exact test to explore patterns in topics by illness phase and topics by creators.

Primary therapy was the most common illness phase (75%), and friend or family of the child with cancer was the most common campaign creator (76%). In 30/76 friend or family creators, a parent posted updates. The most common financial stressors were deductibles/co‐pay costs (60%), employment disruptions (58%), and treatment‐related travel (47%). Nearly all campaigns mentioned non‐financial stressors including describing the illness (88%) and the path to diagnosis (86%). Most campaigns included a tertiary appraisal of worthiness to explain why their child (75%) or the family (49%) was worthy of donations. Most campaigns provided emotional support (84%), few provided peer support (22%), and even fewer provided informational support (10%). There were many significant differences in topics by type of creator and few by illness phase.

**Conclusions:**

Friends and family are most active in creating campaigns near the beginning of a child's cancer journey. Campaigners share information both to update and to convince donors to support them. Future work should explore the consequences of parents engaging in MCF.

## 1 Background

Medical crowdfunding (MCF), fundraising online to mitigate costs of medical care [1, 2], is a customary financial coping behavior in the context of serious chronic illness given the high, ever rising costs of medical care [3] and daily living alongside declining income due to employment disruptions [4, 5]. Most previous studies of MCF have been for adults diagnosed with cancer [6]. The most common reasons for MCF on behalf of individuals with serious chronic illnesses included paying out of pocket (OOP) expenses (deductibles, co‐pays, treatment‐related travel, and lodging) and affording basics such as food and housing [6]. Much of the previous work on MCF has focused on financial outcomes related to individual campaigns. For instance, most recently, a review of MCF literature completed by Cai et al. found that individuals in rural areas, who belong to a racial minority or are women or gender diverse, raise fewer funds than individuals who live in an urban area, are non‐Hispanic white, or men [7]. Furthermore, broadly speaking, MCF does not act as a bridge to provide the necessary resources to those with the greatest need. Rather, it reinforces and exacerbates existing disparities regarding access and available resources [7]. However, a key gap in the current literature is a lack of exploration of the content or topics addressed in the narratives provided on these MCF campaigns.

Like adult cancer populations, families of children (ages 0–17 years) diagnosed with cancer confront substantial financial burdens [4, 8]. Many survivors of pediatric cancer experience material, behavioral, and psychological financial hardships related to cancer treatment. Survivors have expressed concerns about being able to pay for follow‐up treatment, have skipped follow‐up treatment, and costs have impacted their overall financial well‐being [9]. More specifically, the direct costs of food, transportation, childcare for siblings, and employment disruptions to parents or caregivers create substantial financial hardship for families of children with cancer [10]. The impact of these stressors can be conceptualized through the lens of Lazarus and Folkman's Stress, Appraisal, and Coping model [11], and Santacroce and Kneipp's conceptual model of financial toxicity in pediatric cancer [12]. In this context, stressors include the diagnosis of cancer and subsequent costs associated with this diagnosis. A series of appraisals follows, where the adult financially responsible for the child, typically the parents, appraises whether the costs associated with treatment present a threat (i.e., primary appraisal). If so, secondary appraisal considers whether the parent has the needed resources within themselves or their social environment to deal with this threat. These appraisals can trigger financial distress and thus financial coping strategies, such as MCF. A third appraisal relevant to MCF in pediatric cancer is appraisal of worth [13, 14]. This tertiary appraisal involves reflection about whether the child and/or family are worthy of financial support from social network members through MCF.

Evidence of stressors, appraisals, and additional attributes related to MCF (e.g., social support) are found in the online talk on MCF campaign webpages. “Online talk” is text on social media platforms that shares or discusses information in conversational ways [15]. Sources of online talk in MCF campaigns include the introductory narrative, which explains the financial and often the medical situations of the campaign beneficiary, the relationship between the beneficiary and person creating the campaign (“campaigner”), and where the child is in their cancer trajectory, that is, their illness phase. Another source of online talk includes notes donors leave alongside their donation. Examining the online talk publicly available on MCF websites allows for inquiry into stressors, appraisals, and additional attributes of MCF without further burdening caregivers of children with cancer.

Several studies on MCF for serious chronic illness include samples of campaigns that found that anywhere from a quarter to half of their sample had stage IV or end‐stage disease [6]. Given the high overall survival rate for pediatric cancer (~85%; [16]), a more varied distribution of illness phases may be reflected in pediatric cancer‐related MCF campaigns. Describing trends among those phases as financial need inflection points is integral to informing the design of future interventions, including guidance to clinicians on how best to provide anticipatory guidance about MCF and other financial coping strategies.

The purpose of this study is to describe the MCF beneficiaries' illness phases, campaigners' relationships to beneficiaries, and topics of online talk to inform the development of future supportive care interventions to address financial needs in the pediatric cancer context. Understanding critical needs shared on MCF, the timing of needed support, and models of social network involvement are key components that will inform the essential ingredients of future supportive care interventions. The main research questions are: what are the distributions of *illness phases* at campaigns' initiation, *types of relationships* between campaigners and beneficiaries, and *topics of online talk* in MCF campaigns? Exploratory secondary questions are: do distributions of online talk topics differ by (a) illness phase and (b) campaigner type?

## 2 Methods

### 2.1 Sample and Sampling

The analytic sample included public GoFundMe.com (GFM) MCF campaigns to benefit families with a child diagnosed with cancer. Data were derived from this publicly available resource (GoFundMe.com). Inclusion criteria included campaigns (a) written in English, (b) created for a child with cancer ages 0–17 years residing in the United States (US), (c) initiated in the past 3 years, and (d) included a narrative. The search on GFM identified *n* = 1000 such campaigns, and the analytic sample included *n* = 100 of these campaigns. Random sampling was employed to create a representative sample.

The GFM website was searched using the term “childhood cancer” in August 2023. To identify eligible campaigns, over 700 campaigns were screened in a random order over 5 days on the live GFM website. Most campaigns were excluded because their purpose was to raise funds for charity or children living in countries outside the US. Once campaigns were deemed eligible, narrative text written by campaigners, comment text written by donors, and campaigns' demographic information were transferred into Microsoft Word documents. Once campaigns were identified as eligible for inclusion in the analysis, a study ID number was assigned (001‐100). No personally identifiable information (names, dates, geographic locations) was extracted from GFM. This process was informed by guidance from the local Institutional Review Board and was approved through expedited review (IRB; #21‐0907), with a waiver for consent given that the data is publicly available.

### 2.2 Data Collection and Management

Data extracted from campaigns includes fundraising goal, funds raised, number of donors, number of comments, region of campaign, and date initiated. Index child demographics included age at diagnosis and at data collection in years, gender, insurance type, diagnosis, and diagnosis date. Secondly, data on campaigner‐child relationship, illness phase at campaign initiation and data collection, and online talk was extracted from campaigns. Illness phase included primary therapy (treatment for a primary cancer); treatment for high‐risk, progressive, or relapsed disease indicative of treatment intensification; survivorship; and end‐of‐life (EOL) or bereavement. Campaigner types included (a) friends or family, (b) friends or family with parent updates, (c) parents, or (d) unable to determine. Lastly, categories of “topics of talk” were informed by prior qualitative work that found examples of topics to include the following categories: stressors, stress appraisals, appraisals of worthiness, and social support. These pre‐determined categories were coded in Atlas.ti (Berlin, Germany). The process for extracting online talk included two independent coders: (a) reading campaigns one by one in their entirety, (b) writing a case summary, (c) deductive coding using pre‐defined categories, and (d) reading the campaign a third time while adding assigned codes to the Microsoft Excel extraction tool for analysis, and (e) meeting to discuss any discrepancies in coding decisions.

### 2.3 Statistical Analysis

Descriptive statistics were calculated through means/medians, standard deviations/interquartile ranges, and ranges or through frequencies and percentages, as appropriate, to portray the distribution of campaign characteristics, index child demographics, categories of campaign creator‐beneficiary relationships, illness phases, and topics of online talk dichotomized as “yes” if the topic was mentioned or “NM” if not mentioned in the data from GFM. Bivariate relationships between categories of illness phases at campaign creation and online talk, and then between creator‐beneficiary relationships and categories of online talk, were evaluated through Fisher's exact tests using SAS software (version 9.4, Cary, NC). Significance was set at the two‐sided 0.05 level. The contingency tables addressing the “illness phase” question explored the first topic of talk, where columns were mentioned yes/NM (separately for each topic), and rows represented categories of illness phases. Talk topics were then collapsed into fewer, more general categories. These categories were specific financial stressors, general financial stress, primary appraisal, gratitude, offline fundraising, appraisal of worth, tangible support, context on pediatric cancer, informational support, kind words, prayers, emotional support, peer support, sensitive information, and good news shared. This same process was repeated addressing the creator‐beneficiary relationship types. Percentages reported refer to the ratio of the number of mentions of the topic to the total number of campaigns within either the illness phase or the type of campaigner.

## 3 Results

### 3.1 Campaign Characteristics

The median number of donations was 79.5 (IQR: 172.25); most campaigns had between zero and 99 donations (57/100; 57%). Overall, few donors provided comments; the median number of comments was three per campaign (IQR: 10). Seventy‐four campaigns had 10 or fewer comments (74/100; 74%), and 86 campaigns had 20 or fewer (86/100; 86%). Across campaigns, 1 in 27 donors made a comment. The greatest number of comments was 244 (1/100; 1%). Most campaigns had been created within the year prior to data collection (66/100; 66%). See Table 1.

**TABLE 1:** Characteristics of campaigns and beneficiaries.

Demographics	Median (range, IQR)
Amount raised	$6916.50, $0–181 185 (16 354)
Goal amount	$15 000, $100–250 000 (25 000)
Number of donations	79.5, 0–1900 (172.25)
Number of comments	3, 0–244 (10)
Campaign launch date	Count (frequency)
Within 1 year (8.2021–8.2022)	66 (66%)
Within 2 years (8.2020–7.2021)	20 (20%)
2 or more years (before 8.2020)	14 (14%)
Child age at diagnosis	3 years old (2 months–16 years, 5.25)
Child age at data collection	5 years old (0.42–17 years, 8)
Child gender	Count (%)
Male	50 (50%)
Female	47 (47%)
Unable to assess	3 (3%)
Diagnosis	
Solid tumor	55 (55%)
Leukemia or lymphoma	31 (31%)
Brain tumor	9 (9%)
Unable to assess	4 (4%)
Insurance status	
Insured	27 (27%)
Not insured	0 (0%)
Unable to assess	73 (73%)
Insurance type	
Private	8 (8%)
Public	0 (0%)
Unable to assess	92 (92%)
US region of residence	
South	35 (35%)
West	27 (27%)
Midwest	23 (23%)
Northeast	15 (15%)

### 3.2 Index Child Demographics

The median age of the index children at diagnosis was 3 years old (IQR: 5.25). The median age at data collection was 5 years old (IQR: 8). Forty‐seven children were female (47/100; 47%) and 50 were male (50/100; 50%). Gender was assessed via the pronouns used by the campaigner and therefore may not reflect an accurate portrayal of beneficiaries' genders. Most children (70/100; 70%) were diagnosed during the COVID‐19 pandemic, and the predominant diagnosis was solid tumor (55/100; 55%). Information about insurance was difficult to identify (73/100; 73% unable to determine; 92/100; 92% campaigns unclear about private or public). All regions of the US, as informed by the US Census regions, were present in the analytic data set. See Table 1.

### 3.3 Topics of Online Talk

Campaigns (*n* = 100) discussed financial and nonfinancial stressors. The most frequently mentioned stressors were the path to diagnosis (86/100; 86%), the illness itself (88/100; 88%), employment disruptions (58/100; 58%), and out‐of‐pocket costs (75/100; 75%). Out‐of‐pocket costs included deductibles or co‐pays (60/100; 60%) and travel‐related costs (47/100; 47%). More campaigns provided evidence of secondary appraisal (availability of needed resources; 95/100; 95%) than primary appraisal (threat; 56/100; 56%). The most common expressions of secondary appraisals were statements of gratitude for support from their social network (78/100; 78%) and requests that network members donate what they could (71/100; 71%). The most common manifestations of primary appraisal were statements of need for funds (56/100; 56%), and occasionally (5/100; 5%) that the need was urgent. Appraisals of worth were evident in most campaigns (85/100; 85%), most commonly the worthiness of the child (75/100; 75%), followed by the worthiness of the family (49/100; 49%).

Emotional support was often seen in the online talk (84/100; 84%), primarily in the comments (*n* = 76 campaigns included comments). Emotional support found written in the comments by donors included kind and supportive statements from donors or prayers (76/100; 76%) or campaigner requests for prayers/kind thoughts for the family (63/100; 63%). Other common attributes seen in online talk written by campaigners or parent updates were campaigner mobilization of donor support (64/100; 64%), providing information about pediatric cancer (60/100; 60%), and good news (50/100; 50%) about the child's clinical situation or something special the family did to make memories. See Table 2.

**TABLE 2:** Topics of online talk across 100 campaigns^a^.

Topic of talk	% Yes	Exemplar quote^b^
Description of illness	88%	We saw our child in huge pain and discomfort. She became weaker and even contracted pneumonia (ID 001)
Path to diagnosis story	86%	He had a grapefruit size adrenocortical carcinoma behind his liver and kidneys (ID 004)
*Stressors*		
Out of pocket (OOP): deductibles and co‐pays	60%	Treatments are extremely expensive, with the upcoming new year it means the insurance deductible will have to be met (ID 017)
Diagnosis	59%	They thought it was a rare cancer called synovial sarcoma. As a family we were completely devastated because we didn't expect this. (ID 017)
Employment disruptions	58%	We have both had to quit our jobs to be able to attend appointments and hospital stays. (ID 006)
OOP: travel	47%	It's a 7‐h one way trip. The drives are challenging. It literally takes us all day just to drive. (ID 090)
Living expenses	38%	We need funds to be able to pay for our family's regular monthly expenses. (ID 010)
Long treatment	23%	It is a long and intense treatment for Neuroblastoma because recurrence is likely. (ID 067)
General financial burden	21%	To help ease the stress on her parents, so that the family can focus all their efforts on her treatment, recovery and spending precious time together (ID 014)
COVID‐19	19%	With the pandemic, the hospital has visitor policies that's made it hard for us parents to be 100% there and fully support him emotionally. (ID 015)
Stress of diagnosis on siblings	19%	This was a challenging stage for big brother. The realization of the seriousness of the child's disease was very scary and emotional for him (ID 011)
Caregiving for siblings	18%	Dad has taken time off from work to help with the 2 little ones when she is in the hospital, which happens a lot (ID 092)
Quality of life enhancements	15%	We need help in modifying our bathroom to accommodate her handicap needs. (ID 023)
Demographics factor	8%	There is about $5000–$8000 per year that mom has to pay for out of pocket which is difficult for a single mom. (ID 004)
Burial costs	3%	They are very low income and cannot afford a proper burial and service for their sweet angel. Please help them lay their beautiful boy to rest. (ID 079)
Complementary and alternative medicine	2%	She is treated with cannabis tincture oils that help her get through these awful toxic treatments. (ID 092)
*Primary appraisal*		
Need for funds	56%	Soon we will reach the point where we can no longer stay afloat and afford our family's needs through the remainder of his treatment. (ID 010)
Urgent need for funds	5%	We currently don't have a permanent car to drive to medical appointments and procedures (ID 065)
*Secondary appraisal*		
Gratitude	78%	We don't know how we can express our gratitude for your generosity. Your thoughts, prayers and support have been a bright light in the dark (ID 050)
Donate what you can	71%	We know that times are tough for everyone right now and you may not be able to donate so please don't feel like you have to. Please only donate if you are truly able to (ID 017)
Mention of offline fundraising	40%	[regarding a benefit concert] It fills our hearts to see everyone come to the concert to support her and her dad, thank you for the financial help and the immense love our community has shown us (ID 040)
Donate specific items we've requested	12%	We are hoping to raise funds to buy a more reliable car for the family. (ID 035)
*Appraisal of worth*		
Of the child	75%	He's been doing so well, he has been so happy and continues to tell jokes, which just reminds us how incredibly strong he is (ID 034)
Of the family	49%	Dad is former military and a police officer, and he and his wife have been generous members of their communities for forever, they now need YOUR help. (ID 019)
Of pediatric cancer	11%	So little funding goes to pediatric cancer‐ less than 4%. Drugs that are used now have been used for 60–70 years (ID 075)
*Additional attributes*		
Emotional support	84%	Now this GoFundMe is a place where we can update you all who have been so caring and been a place where you all have generously helped the family in their need because cancer is so challenging in EVERY way, including financially (ID 075)
Kind words said by donors^c^	64%	Please look after yourselves, I'm sorry that life has been so hard on your family but know that you and your child are loved. (ID 071)
Mobilization of support	64%	Please donate blood to a local blood bank if you are unable to donate money. (ID 030)
Asking for prayers	63%	We need a miracle. Please pray for him. (ID 071)
Donors sharing prayers^c^	63%	I don't know you, but my mom fought cancer 3 times and other's generosity was what helped us get through it. Praying for your family. (ID 071)
Context of pediatric cancer	60%	I'd really like you to pause and read and learn. I want to spread awareness of this disease because it isn't as rare as we think it is. (ID 010)
Sharing good news	50%	He went back to school this week, the first time in 2 years! He's also looking forward to rejoining his baseball team. (ID 085)
Receipt of funds	47%	Wow! You all are amazing. We've reached our 2nd goal and you all have helped so much! (ID 090)
Sharing sensitive information	39%	I mean how can you respond when your 6 year old tells you she is so lonely‐ that she has no friends, and chemo won't let her have friends (ID 088)
Faith has helped coping	29%	We try to stay strong in faith and trust in God. We pray he guides her treatment team to make the best decisions for our girl. Faith is what keeps us going. (ID 092)
Peer affirmational statements	22%	Through this GoFundMe I can cope, vent, and cry out to other parents who know what I'm going through. To share with them what I feel. And maybe they have felt the same things. (ID 005) *(beneficiary to donor peers)* We hope this helps. When our son was diagnosed with cancer a few years ago, the generosity we received was so helpful with managing our new normal. (ID 035) *(donor to beneficiary peer)*
Financial resources used	10%	We are trying to get more medical supplies paid for with help from an organization and to see if they can fund Grandma and Auntie as caregivers so Dad can have a break (ID 040)
Mention of religious activities	4%	Our church prayer list has his name on it and I know God is protecting him. I will pray for him. (ID 011)

^a^ Multiple topics were mentioned by a single campaign across the sample.

^b^ Quotes have been modified from their original language, so they are not identifiable through search engines.

^c^ Sample of campaigns that had comments posted from donors: *n* = 76.

### 3.4 Illness Phases

Primary therapy was the most prevalent illness phase at campaign initiation (75/100; 75%). Sixteen campaigns were for children in the treatment intensification phase (16/100; 16%), two were for children in survivorship (2/100; 2%), five were for children at EOL or families in bereavement (5/100; 5%), and two campaigns had no information about illness phase (2/100; 2%). Sixteen campaigns (16/100; 16%) encompassed multiple illness phases. See Table 3.

**TABLE 3:** Frequencies of mentioned topics of talk by illness phase.

Topic of talk		Primary therapy *n* = 75	Intensified therapy^b^ *n* = 16	Survivorship *n* = 2	EOL^c^ *n* = 5	UTA^d^ *n* = 2	*p*
Specific financial stressors *n* = 89	Count	66	16	2	4	1	0.17
	% within type^e^	88.0	100.0	100.0	80.0	50.0	
General financial stress *n* = 21	Count	17	2	0	2	0	0.67
	% within type	22.7	12.5	0	40.0	0	
Primary appraisal *n* = 56	Count	41	10	2	3	0	0.46
	% within type	54.7	62.5	100.0	60.0	0	
Gratitude *n* = 78	Count	59	13	2	3	1	0.52
	% within type	78.7	81.3	100.0	60.0	50.0	
Offline fundraising *n* = 40	Count	30	10	0	0	0	0.04^a^
	% within type	40.0	62.5	0	0	0	
Appraisal of worth *n* = 85	Count	62	14	2	5	2	0.94
	% within type	82.7	87.5	100.0	100.0	100.0	
Mention of tangible support *n* = 47	Count	36	9	1	1	0	0.51
	% within type	48.0	56.3	50.0	20.0	0	
Context about pediatric cancer *n* = 60	Count	47	10	2	0	1	0.03^a^
	% within type	62.7	62.5	100.0	0	50.0	
Informational support *n* = 10	Count	10	0	0	0	0	0.56
	% within type	13.3	0	0	0	0	
Kind words from donors *n* = 64	Count	48	11	1	4	0	0.40
	% within type	64.0	68.8	50.0	80.0	0	
Prayers from donors *n* = 63	Count	50	10	0	3	0	0.13
	% within type	66.7	62.5	0	60.0	0	
Emotional support *n* = 84	Count	67	12	1	4	0	0.009^a^
	% within type	89.3	75.0	50.0	80.0	0	
Peer support *n* = 22	Count	17	3	0	2	0	0.85
	% within type	22.7	18.8	0	40.0	0	
Sensitive information *n* = 39	Count	28	8	0	3	0	0.45
	% within type	37.3	50.0	0	60.0	0	
Good news *n* = 50	Count	39	10	1	0	0	0.05^a^
	% within type	52.0	62.5	50.0	0	0	

^a^ Indicates significant finding at alpha < 0.05.

^b^ Intensified therapy for high‐risk, progressive, or relapsed disease.

^c^ End‐of‐life or bereavement.

^d^ Unable to assess.

^e^ % within type of illness phase.

### 3.5 Online Talk by Illness Phase

**FIGURE 1:**
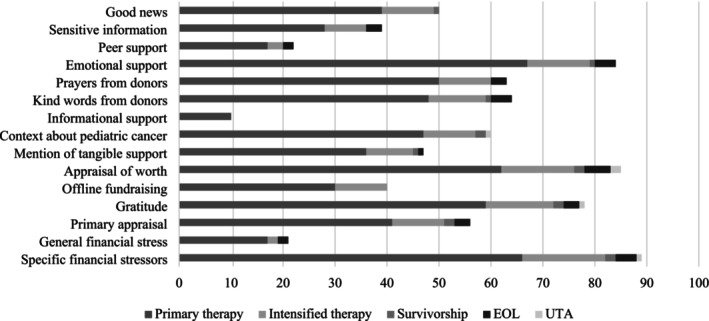
Distribution of mentioned topics of talk by illness phase.

Few statistically significant differences were found in online talk topics by illness phases at campaign initiation. Some differences were observed in whether good news was shared, emotional support, or information about pediatric cancer were provided, and offline fundraising was mentioned among the different illness phases (all *p* < 0.05). For all remaining topics of talk, no statistically significant differences were identified regarding whether the topic was mentioned among the observed illness phases (See Figure 1).

Among campaigns benefiting children in primary therapy (39/75; 52.0%) or treatment intensification phase (10/16; 62.5%) had more mentions of good news than no mentions. Among campaigns for children in the EOL/bereavement phase, none mentioned good news (0/2). Across phases, most campaigns (*n* = 84/100, 84%) mentioned emotional support. Most campaigns for children in primary therapy (47/75; 62.7%) or intensification phases (10/16; 62.5%) provided contextual/educational information on pediatric cancer, while none for children at EOL did so. Offline fundraising was not mentioned by any EOL campaigns, and only 40% (30/75) of campaigns for children in primary therapy did so, whereas the majority (63%, 10/16) of campaigns for children in the intensification phase mentioned offline fundraising. See Table 3.

### 3.6 Campaigner‐Beneficiary Relationships

Most campaigners were female (87/100; 87%) and friends or family members (76/100; 76%). Parents provided updates in 30 of 76 (39.5%) campaigns created by others and were the sole campaigners for 19% (19/100) of campaigns. Relationships to beneficiaries could not be discerned for 5% of campaigns (5/100). See Table 4.

**TABLE 4:** Frequencies of mentioned topics of talk by campaigner type.

Topic of talk		Parent *n* = 19	FPU^b^ *n* = 30	FF^c^ *n* = 46	UTA^d^ *n* = 5	*p*
Specific financial stressors *n* = 89	Count	17	28	40	4	0.56
	% within type^e^	89.5	93.3	87.0	80.0	
General financial stress *n* = 21	Count	2	3	15	1	0.06
	% within type	10.5	10.0	32.6	20.0	
Primary appraisal *n* = 56	Count	12	15	28	1	0.30
	% within type	63.2	50.0	60.9	20.0	
Gratitude *n* = 78	Count	16	28	31	3	0.02^a^
	% within type	84.2	93.3	67.4	60.0	
Offline fundraising *n* = 40	Count	9	17	13	1	0.06
	% within type	47.4	56.7	28.3	20.0	
Appraisal of worth *n* = 85	Count	16	29	36	4	0.12
	% within type	84.2	96.7	78.3	80.0	
Mention of tangible support *n* = 47	Count	10	20	16	1	0.03^a^
	% within type	52.6	66.7	34.8	20.0	
Context about pediatric cancer *n* = 60	Count	14	22	22	2	0.06
	% within type	73.7	73.3	47.8	40.0	
Informational support *n* = 10	Count	1	5	4	0	0.59
	% within type	5.3	16.7	8.7	0	
Kind words from donors *n* = 64	Count	13	25	25	1	0.01^a^
	% within type	68.4	83.3	54.4	20.0	
Prayers from donors *n* = 63	Count	7	25	30	1	0.001^a^
	% within type	36.8	83.3	65.2	20.0	
Emotional support *n* = 84	Count	15	29	39	1	0.001^a^
	% within type	79.0	96.7	84.8	20.0	
Peer support *n* = 22	Count	3	11	8	0	0.14
	% within type	15.8	36.7	17.4	0	
Sensitive information *n* = 39	Count	11	18	9	1	0.0005^a^
	% within type	57.9	60.0	19.6	20.0	
Good news *n* = 50	Count	12	26	11	1	< 0.0001^a^
	% within type	63.2	86.7	23.9	20.0	

^a^ Indicates significant finding at alpha < 0.05.

^b^ Friend or family with parent update.

^c^ Friend or family.

^d^ Unable to assess.

^e^ % within type of campaigner or % within illness phase.

### 3.7 Online Talk by Campaigner Type

**FIGURE 2:**
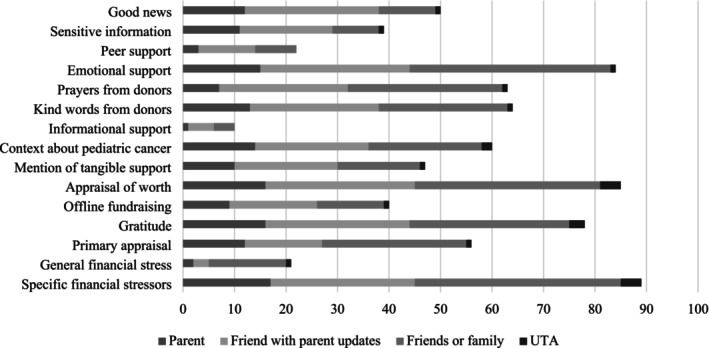
Distribution of mentioned topics of talk by campaigner type.

Statistically significant differences were found in online talk by campaigner type. Some differences were observed in whether the following topics were mentioned: good news, sensitive information, emotional support, kind comments from donors, prayers from donors, mention of tangible support, and gratitude (all *p* < 0.05). There was no difference in whether the remaining topics of talk were mentioned among the different types of campaigners (see Figure 2).

Good news was mentioned in most campaigns written by family or friends' campaigns with parent updates (26/30; 86.7%), and in campaigns by parents (12/19; 63.2%). Conversely, most campaigns by friends or family *without* parent updates lacked mentions of good news (35/46; 76.1%). Sensitive information was mentioned among most friends or family with parent updates campaigns (18/30; 60.0%) and many parent campaigns (11/19; 58.0%). Friends or family members *without* parent updates campaigns typically did not mention sensitive information (37/46; 80.4%). Across all categories of relationships between campaigners and the index child, campaigns typically mentioned emotional support. Nearly all campaigns by friends or family with (25/30; 83.3%) and without (30/46; 65.2%) parent updates mentioned donors praying for the family, whereas most campaigns written by parents did not (12/19; 63.2%). Kind words from donors and expressions of gratitude were common across all types of campaigners. See Table 4.

## 4 Discussion

Key findings from this study include that the most common topics of talk represented in the random sample of GFM campaigns were nonfinancial about the child's illness and financial costs of out‐of‐pocket expenses and employment disruptions. Friends and family were the most common campaigners, and the most common illness phase was primary therapy. While few categories of online talk differed significantly by illness phase, most topics differed significantly by campaigner. The most mentioned financial stressors were cost‐sharing, parental employment disruptions, and treatment‐related travel. Campaigners articulated secondary appraisal (that the social network members had the resources needed by the index child's parents to cope with financial threats) through expressions of gratitude for that support. Expressions of gratitude were often connected to statements like “donate what you can”, likely due to campaigners and/or parents not wanting to seem greedy. Almost all campaigns included kind words and/or prayers from donors, indicative of emotional support. However, few campaigns mentioned informational support or peer support.

The most common illness phase represented in our analytic sample was primary therapy. This is consistent with prior studies of campaigns for adult or adolescent and young adult (AYA) survivors of cancer [17, 18]. Upon hearing about someone's diagnosis, social network members can feel inclined to do something concrete to support the person and/or their family [17]. Perhaps this is also indicative of social networks being aware of the exorbitant costs of cancer and realizing money will be needed. However, a disconnect may exist when parents believe they do not currently need (or deserve) support near diagnosis, but near diagnosis is when social networks are most active. Previous studies of parents of children with cancer found that perceived social support declines over time. This could be a function of having less time to seek support [19] or exhaustion of the social network. Therefore, parents should be counseled on anticipated costs of treatment and to assess what support they have available to them. Evidence that most campaigns in the intensification phase mentioned offline fundraising whereas that was not observed in other illness phases reinforces this notion to take stockpile support when it is available. This finding implies that even with online fundraising, the need for financial (and possibly emotional) support among families whose child has relapsed or had treatment intensification goes beyond what is achievable online.

That most campaigners are friends or family, along with most campaigns are in the primary therapy illness phase, may further suggest that support is more readily available early in the cancer trajectory. While others have found that friends or family are usually the campaigners [6], most prior studies did not account for the likelihood of multiple authors on campaign pages—including the patient or the patient's caretaker/parent. This suggests that having friends or family members lead the MCF endeavor does not eradicate parental effort. Assessing parents' involvement in MCF may be important because the highest percentages of talk topics were in the friend or family with *parent* updates campaigner category. While parents likely appreciate assistance with tangible support and organizing for them [20], they are responsible for providing day‐to‐day updates to engage social network members and generate donations.

The utility of this parental involvement cannot be determined from this study. The campaign may provide a venue in which parents can broadly share information and/or that this writing is a way to share their hopes, fears, and frustrations [21]. Alternatively, the writing could contribute to parental overwhelm, another task on a never‐ending to‐do list amidst physical and emotional exhaustion. Future work should elucidate the utility of parental involvement in campaigns.

The study results also suggest campaigners' awareness, both in their descriptions of the child and the family (appraisal of worthiness) and their secondary appraisals, of how their writings would be perceived by potential donors. Specifically, the proportion of campaigners who wrote something like “donate what you can” implies that campaigners believed that donors while couldn't be expected to cover medical care or replace lost income, any help was desperately needed. Prior studies found that donors evaluate need when reading campaigns and that the campaigner's justification of worthiness is key [22]. However, justification of worthiness may come with socio‐emotional costs to the parent. Future work should explore these socio‐emotional costs and how they affect the utility of MCF. Similarly, many campaigners wrote words of gratitude or thanks for the donations that were received. The implications of this finding are two‐fold: mitigating a misconception of greediness and also the practical application of communicating in a one‐to‐many format. First, expressions of gratitude could have been related to the justification of worthiness. Campaigners may have felt that they were responsible to the donors and needed to update them on the progress of the campaign while simultaneously thanking their social network that had already donated. Alternatively, these expressions of gratitude could be a functional tool for campaigners to provide words of thanks to donors without having the time and emotional burden of contacting each donor individually. It is probable that both these applications occurred at different times for campaigners and depending on the situation may be reduced or increased perceived stress.

A potential mechanism through which MCF could mitigate childhood cancer‐related stress is exchanges of emotional support via MCF. Most campaigns in this sample included kind words or prayers from donors that varied in quantity and skewed toward fewer rather than more comments (median = 3 comments; IQR = 10). It was outside the scope of this study to examine the relationship between length of campaign and number of comments (and content of those comments), but it is an important area of future work. Furthermore, future research of these types of comments may shed light on how campaigns function as a venue for emotional support. However, there may be limitations to the emotional support that can be exchanged on MCF. For instance, only those that can donate funds on GFM can post words of support—thus limiting the pool of supporters that can be active on this website. Additionally, the number of comments may be less important than the number of donations. Seeing people show up to donate, whether they commented, may be the more highly valued form of support. Informational support and peer support were not often observed in the analytic sample. Prior studies that used Facebook had similar findings. In a study of social support for pediatric cancer caregivers that used Facebook, less than 1% of examined posts expressed informational support from experiential peers [23, 24]. However, uncommon is not equivalent to unimportant. Future research of parents about their views on successful campaigns and the support they did or did not receive could help explain these findings to inform intervention development.

## 5 Clinical and Policy Implications

That nonfinancial topics were most referenced, that friends or family being the most cited campaigners, and that campaign initiation most often occurred in the primary illness phase suggests that systematic screening for social support beginning at diagnosis is integral to obtaining information about financial hardship experienced by families. Identifying families who lack social networks that can engage in MCF campaigns and share information about non‐financial illness‐related stressors could influence their ability to cope with stressors inherent in their child's illness, and thus their health and wellbeing. Furthermore, future research into examining beneficiaries' appraisal of MCF and whether having this financial and emotional support was protective will inform how clinicians should approach conversations about MCF with families. Likewise, the topics discussed on these campaigns, particularly those related to financial (medical expenses, employment disruptions, travel costs, living expenses), are crucial areas for local and national policy. Institutional policy, such as free parking, could be a major relief to many families that are struggling with the travel expenses related to their child's treatment. Given that many campaigns were created within the primary treatment illness phase, it is clear that these financial needs develop quickly after diagnosis. Therefore, national policy that includes caregiver employment protection and the ability to earn an income while acting as a caregiver is needed policy initiatives.

### 5.1 Study Limitations

Overall, these findings add to knowledge about MCF in pediatric cancer and can inform inflection points for interventions. However, the findings may not generalize to families not participating in MCF and are perhaps biased toward parents who have robust social networks with discretionary wealth to share [25]. Additionally, this sample may be biased toward those that either have someone in their social network that is comfortable with sharing information online, and/or that the beneficiary family is comfortable, or at least willing, to share information in a public format. It is also important to note that families utilizing fundraising are not limited to only GFM; the data available for this study does not encompass all types of fundraising efforts, and additional insights into stress and support should be studied in fundraising outside of online crowdfunding platforms like GFM. There are also limitations to what can be concluded from the topics of talk. Given that the topics were assessed from previously written campaigns and there was no contact with campaigners or beneficiaries, we could not conclude whether a topic was not relevant to the campaigner and/or beneficiary, only that they chose not to mention it in their campaign. Additionally, analyzes were limited based on sample sizes, particularly for some of the topics of talk not having sufficient observations. Future work with beneficiaries and campaigners can help fill in this gap and confirm findings regarding stressors, appraisal, and additional attributes.

This study presents descriptions of what campaigners choose to share about their financial and nonfinancial stressors with the public. Future studies should further explore perceptions and experiences of parent beneficiaries of MCF and link MCF to socio‐emotional outcomes. Future studies should also examine the utility of MCF, explore the contributions of MCF to medical and quality of life outcomes, and identify key benefits gained through MCF that can be replicated in other types of financial distress interventions.

## 6 Conclusion

Evidence of financial and non‐financial stressors, appraisal of worth, expressions of gratitude, and presence of emotional support through kind words and prayers from donors were commonly observed across MCF campaigns for children diagnosed with cancer. Friends and family typically create the campaign, mainly when the child is starting the primary treatment phase. Future studies should examine beneficiary perceptions of MCF and uncover what key features of crowdfunding to include in interventions to mitigate the financial burden on families experiencing pediatric cancer.

## Author Contributions

**Mary K. Killela:** conceptualization, data curation, formal analysis, investigation, methodology, project administration, writing – original draft, writing – review and editing. **Sandra Garcia:** formal analysis, project administration, writing – review and editing. **Jessica Keim‐Malpass, Sandra Soto, Todd Schwartz, Jessica Williams, Sheila Santacroce:** conceptualization, supervision, writing – review and editing.

## Conflicts of Interest

The authors declare no conflicts of interest.

## Data Availability

The data that support the findings of this study are available from the corresponding author upon reasonable request. The data were derived from publicly available sources.
